# Systematic Review of miRNA as Biomarkers in Alzheimer’s Disease

**DOI:** 10.1007/s12035-019-1500-y

**Published:** 2019-02-08

**Authors:** S. Swarbrick, N. Wragg, S. Ghosh, Alexandra Stolzing

**Affiliations:** Centre for Biological Engineering, Wolfscon School of Mechanical, Electrical and Manufacturing Engineering, Loughborough University, Loughborough, UK

**Keywords:** miRNA, Alzheimer disease, Biomarkers, Brain, Peripheral blood

## Abstract

Currently there are 850,000 people with Alzheimer’s disease in the UK, with an estimated rise to 1.1 million by 2025. Alzheimer’s disease is characterised by the accumulation of amyloid-beta plaques and hyperphosphorylated tau in the brain causing a progressive decline in cognitive impairment. Small non-coding microRNA (miRNA) sequences have been found to be deregulated in the peripheral blood of Alzheimer patients. A systematic review was conducted to extract all miRNA found to be significantly deregulated in the peripheral blood. These deregulated miRNAs were cross-referenced against the miRNAs deregulated in the brain at Braak Stage III. This resulted in a panel of 10 miRNAs (hsa-mir-107, hsa-mir-26b, hsa-mir-30e, hsa-mir-34a, hsa-mir-485, hsa-mir200c, hsa-mir-210, hsa-mir-146a, hsa-mir-34c, and hsa-mir-125b) hypothesised to be deregulated early in Alzheimer’s disease, nearly 20 years before the onset of clinical symptoms. After network analysis of the 10 miRNAs, they were found to be associated with the immune system, cell cycle, gene expression, cellular response to stress, neuron growth factor signalling, wnt signalling, cellular senescence, and Rho GTPases.

## Dementia

Dementia is a common syndrome in people over 65 years of age and is characterised by a progressive decline in memory and other abilities [1]. In 2014, there were 850,000 people living with Alzheimer’s disease in the UK, costing the economy £26.3 billion a year. Due to the ageing population, this is set to rise to over 1.1 million people with Alzheimer’s disease in 2025 [2].

The Prime Ministers Challenge on Dementia 2020, established by the UK Government, found in 2010/11 that only 42% of estimated dementia sufferers in England had a formal diagnosis [3]. In 2016, the diagnosis rate increased to 67% [4]. This has been attributed to an increased public awareness of dementia, a reduction in the stigma associated with dementia, and an increase in dementia research. Due to the nature of dementia, the accuracy of the diagnosis correlates with the severity of the symptoms and varies from 9 to 41% [4]. Onset of dementia can occur 20–30 years before the first symptoms appear [5]; therefore, the earlier a diagnosis can be made, the less developed the degeneration will be, increasing the probability of a successful treatment.

### Alzheimer’s Disease

**Fig. 1:**
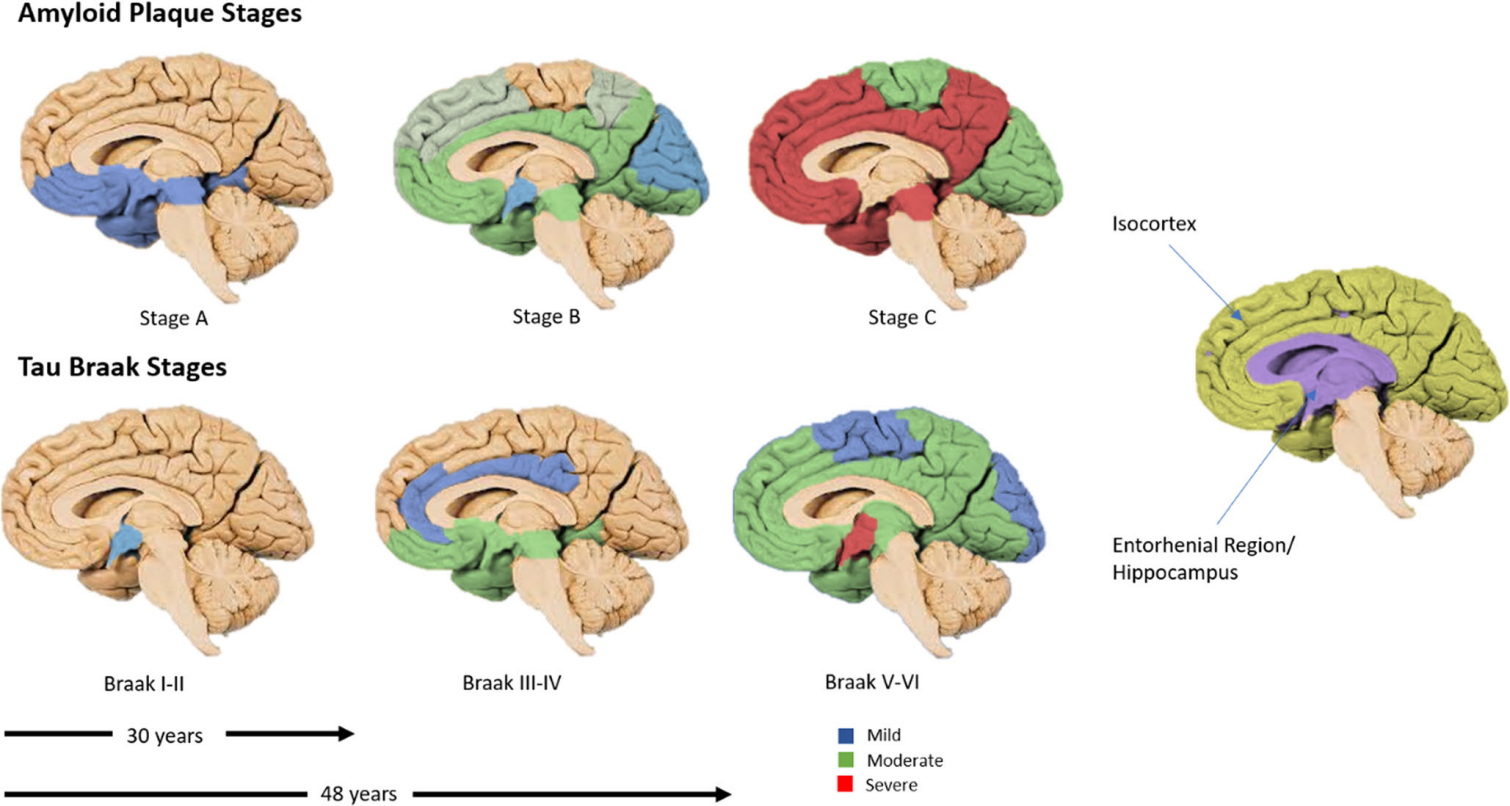
Schematic of the Braak and Braak amyloid and tau stages during the progression of Alzheimer’s disease. Mild, moderate, and severe correspond to the density of amyloid/tau protein

Alzheimer’s disease is the most common form of dementia accounting for 62% of the dementia patients [2] and is characterised by the presence of amyloid plaques and hyperphosphorylated tau in the brain. In 1991, Braak and Braak mapped the movement of both amyloid-β and hyperphosphorylated tau in the brain during the progression of the disease [6]. The movement of amyloid was split into three Stages (A–C) and that of tau into six (I–VI), as shown in Fig. 1.

Amyloid deposits are mainly found in the isocortex of the cerebral cortex. The plaques are not uniform in shape or size and early stage accumulation suffers from inter-individual variation. The amyloid deposition develops before the onset of tau. However, the presence of amyloid does not mean that tau pathology will develop [6]. During Stage A, amyloid is found in the base layer of the frontal, temporal, and occipital lobes. In Stage B, amyloid progresses to almost all isocortex areas and during Stage C, amyloid becomes densely packed [6].

Tau Braak Stages correlate with the progression of Alzheimer’s disease. It is estimated that it can take 48 years to develop from Braak Stage I to Braak Stage V in which Alzheimer’s disease symptoms are apparent. A large proportion of that time is when the disease is non-symptomatic as it can take 30 years to progress from Braak Stage I to Stage III [5].

Braak Stages I and II are centred around the transentorhinal region with Stage II being more densely packed with tau pathology than Stage I. At Stage III, the pathology moves into the entorhinal region with low levels of tau seen in CA1 of the hippocampus and no or mild changes present in the isocortex [6]. The hippocampus is responsible for episodic memory, which is memory of autobiographical events [7]. This corresponds with early symptoms seen in Alzheimer’s disease and is defined as mild cognitive impairment (MCI). Patients that fit this definition are 3–5 times more likely to develop dementia within 3–5 years [8].

At Stage IV, there is increased pathology in the entorhinal region and CA1 hippocampus. At this stage, there is no detectable brain atrophy, and the pathology does not meet the criteria for neuropathologic diagnosis of Alzheimer’s disease. At Stage V, tau is found in almost all areas of the hippocampus and isocortex, with the areas becoming severely affected by Stage VI. Involvement of the isocortex corresponds to late Alzheimer’s disease and clinical diagnosis [6].

Alzheimer disease symptoms have been classified by criteria published in 1984 by both the National Institute of Neurological and Communicative Disorders and Stroke (NINCDS) and the Alzheimer’s Disease and Related Disorders Group (ADRDG). It concludes that a definitive diagnosis can only be given when histological analysis by biopsy or autopsy has been conducted [1]. If a biopsy cannot be conducted, then a possible or probable diagnosis is given. A probable diagnosis has a sensitivity of 81% and specificity of 70%, a possible diagnosis has a sensitivity of 93% and specificity of 48% [9]. Sensitivity is the ability to distinguish between normal and Alzheimer’s disease, while specificity is the capability to differentiate Alzheimer’s disease from other types of dementia.

### Diagnostic Techniques for Alzheimer’s Disease

An ideal diagnostic technique for Alzheimer’s disease would be that which can identify the disease with adequate reliability considerable time before the onset of symptoms for treatments to be effective, and which is minimally invasive, low-cost, and easy to be applied for mass screening. Current diagnostic techniques for Alzheimer’s disease primarily include cognitive testing [10], neuroimaging [11], and biomarker detection [12]. Other more recently reported diagnostic tests include retinal imaging of amyloid beta, structural changes in the retina [13, 14], and alterations in an Alzheimer patient’s sense of smell [15]. Cognitive testing, for example questionnaires like the mini mental state examination (MMSE), is the most commonly used tool to asses a patient’s symptoms for Alzheimer’s disease [16]. Therefore, cognitive testing is unable to diagnose the disease in the pre-symptomatic stage [17]. Neuroimaging diagnosis, for example magnetic resonance imaging, looks for hippocampal atrophy [18]. However, this is an expensive and specialised technique, which is logistically challenging to be used for mass screening.

Detection of biomarkers in patients is heavily reported for cerebrospinal fluid (CSF) and peripheral blood [references]. Other biological samples, such as urine [19], breath [20], and saliva [21, 22], have the potential for biomarker detection although they are less prominent in the literature.

CSF requires an invasive lumbar puncture procedure under general anaesthetic with common side effects including mild to moderate headache in up to 46% of cases [23–25]. The most commonly reported CSF assay looks for a decrease in amyloid-beta 42 and increased levels of total tau and phosphorylated tau. The test’s sensitivity ranges between 68 and 95% and specificity between 83 and 97% [26–30]. To quantify concentrations of amyloid beta and tau, studies use enzyme-linked immunosorbent assays (ELISAs). Multi-centre studies conducted using ELISAs have demonstrated a large variability in results [31]. Currently, this variation remains too high to establish international cut-off values, which differentiate Alzheimer patients from normal controls [32].

Another approach is to screen for biomarkers in peripheral blood. Blood collection is significantly less invasive than lumbar puncture and routinely conducted. Therefore, detecting biomarkers in peripheral blood is potentially more applicable to mass screening and regular monitoring of disease progression. Several studies have found differences in specific protein and microRNA (miRNA) concentrations between normal and Alzheimer’s disease blood, highlighting its potential as a diagnostic procedure [12, 33–36]. This review will focus on miRNAs only.

## miRNAs

**Fig. 2:**
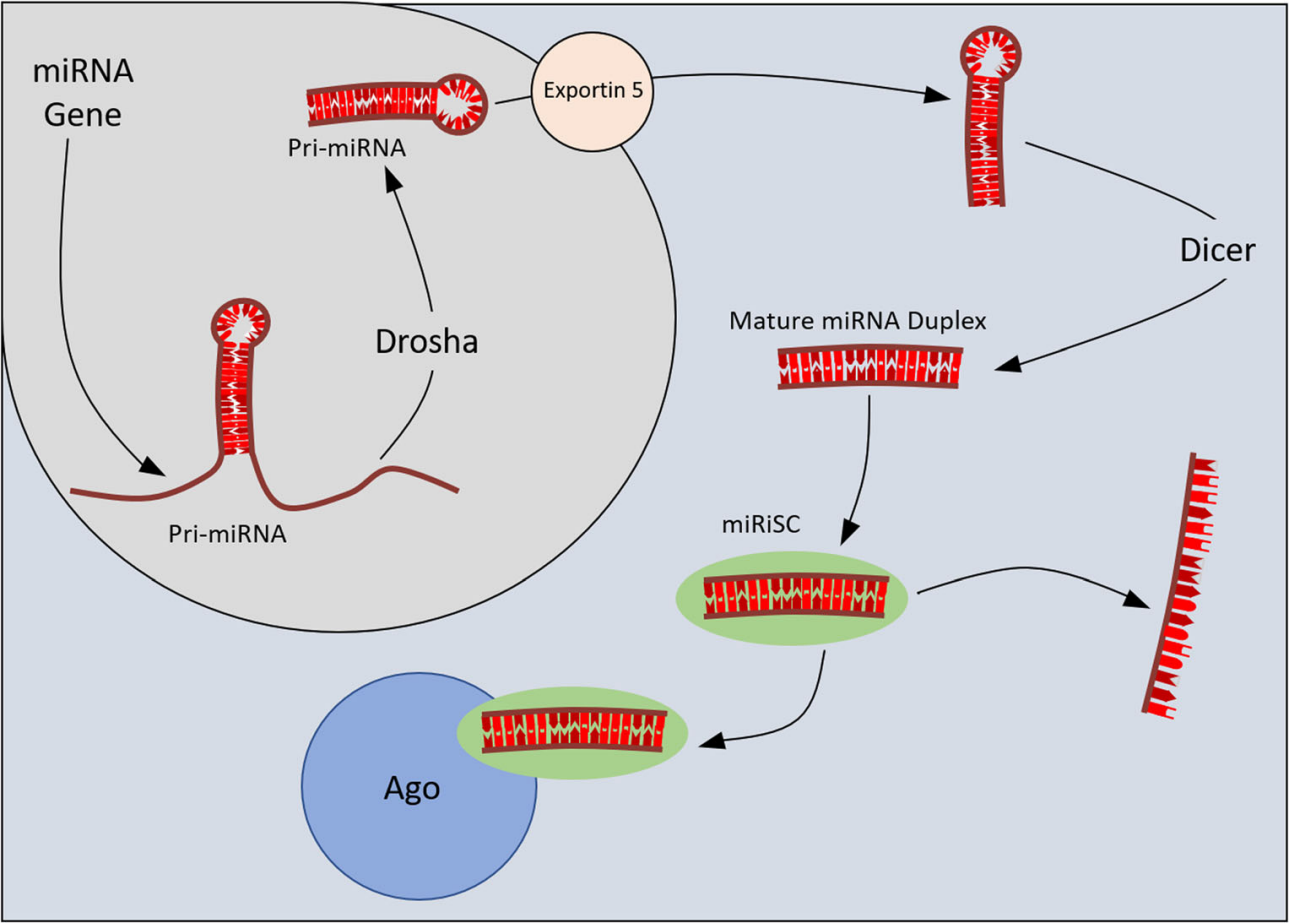
Schematic showing the synthesis of miRNA

miRNA are small non-coding RNA, normally 22–23 nucleotides, that control gene expression by binding to the 3′-untranslated region (UTR) region in messenger RNA (mRNA). Through this, they suppress translation or induce degradation of the target mRNA [37]. miRNAs are transcribed by RNA polymerase II/III in the nucleus to large RNA precursors called pri-miRNA. The pri-miRNA is processed by the RNase III enzyme Drosha to be approximately 70 nucleotides in a hairpin structure. The pri-miRNA is then exported to the cytoplasm by exportin 5. After subsequent processing by the RNase III enzyme Dicer, it releases a small RNA duplex which is then loaded into an Argonaute (Ago) protein. The mature miRNA then directs the Ago-miRNA complex to the target mRNA (Fig. 2) [37–39]. The Ago-miRNA complex is very stable in body fluids, and miRNA can be attributed to specific organs and pathologies, making miRNA an ideal biomarker target [40].

## miRNA Implicated in Alzheimer’s Disease

The literature includes a number of recent studies reporting miRNAs in blood, CSF, or brain as candidate biomarkers for Alzheimer’s disease [references]. Besides variations in the quantification methods and protocols used, the comparability of these studies is particularly challenged due to discrepancies in the stage of Alzheimer’s disease for patients included in the studies. In view of this challenge, the objective of this review was to identify the miRNAs that are deregulated in peripheral blood in late Alzheimer’s and compare them with those found altered in the brain during an early stage of the disease (Braak Stage III). Correlation of deregulated blood-based miRNAs in peripheral blood with those altered in Braak Stage III will allow nearly a 20-year window for screening of patients at risk of Alzheimer’s before the onset of pathological symptoms (Fig. 1).

To establish the number of miRNA found to be significantly deregulated in Alzheimer’s patients, keywords were placed in search databases including Web of Science, Google Scholar, and PubMed. Keywords chosen were “miRNA,” “Alzheimer,” “diagnosis,” and “biomarker” with either “blood,” “serum,” “plasma,” “cerebrospinal fluid,” or “brain.” Both the article title and abstract were assessed for applicability into the review. Last searches were conducted in October 2017. The following inclusion and exclusion criteria were used for the systematic review. The inclusion criteria were as follows:

1. All samples tested were human
2. Aged matched controls were used
3. Articles were in English
4. A sample group of three or more.

Exclusion criteria:

1. Review articles, conference abstracts, and studies without a complete set of data.
2. Articles that do not mention Alzheimer’s or dementia in the title or abstract.

The following information was then extracted from the selected articles: Fist named author, year of publication, participant country, blood sample type used, number of control participants, number of Alzheimer patients, any other participant group used, Alzheimer’s disease diagnostic technique, and the significantly deregulated miRNA.

## miRNA Deregulation in Blood

From the systematic review, 20 articles were found to look at miRNA blood deregulation in Alzheimer patients. Nineteen articles were published between 2012 and 2016 and one in 2007 are summarised in Table 1.

**Table 1:** Summery of articles found after systematic review of miRNA deregulated in the peripheral blood in Alzheimer patients

Total number of articles	20
Year of publication	2012 to 2016 and 2007
Most frequent technique used to diagnose Alzheimer’s disease	12 articles used MMSE
Most frequent miRNA detection technique used	15 articles used PCR

From the 20 articles, 102 miRNAs were found to be deregulated in Alzheimer patient’s blood compared to aged matched controls [41–57]. Ten articles looked at serum blood samples [44–47, 50, 55, 57–60], 4 at plasma [48, 50, 51, 61], 3 at blood mononuclear cells (BMC) [43, 52, 62], 2 in exosomes [42, 49], and 1 in whole blood [41]. This corresponded to 56 miRNAs found to be deregulated in serum, 10 in plasma, 11 in whole blood, 10 in BMC, and 15 in exosomes as shown in Table 2. The highest fold changes were seen in the plasma.

**Table 2:** Number of articles and miRNA found to be deregulated between Alzheimer patients and controls for different blood components

Blood component	Number of articles	Number of miRNA
Serum	10	56
Plasma	4	10
Whole blood	1	11
BMC	3	10
Exosomes	2	15

*BMC*, blood mononuclear cells

**Fig. 3:**
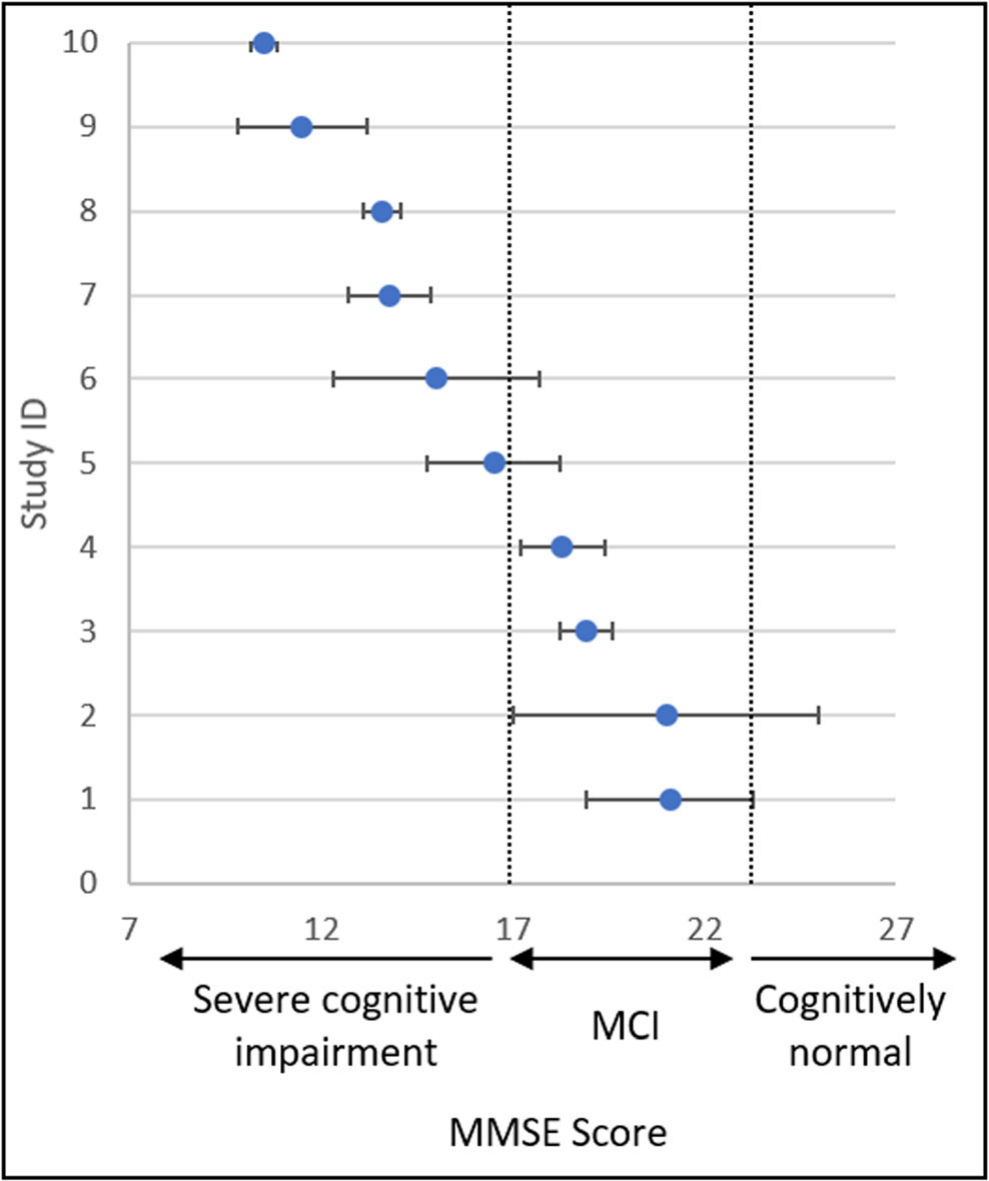
Forest plot showing the distribution of MMSE scores from 10 articles. Study ID: (1) Kiko (2014) [51], (2) Cheng (2014) [42], (3) Leidinger (2013) [41], (4) Zhu (2014) [59], (5) Kumar (2013) [48], (6) Geekiyanage (2012) [56], (7) Wang (2015) [61], (8) Tan (2014) [47], (9) Dong (2015) [57], and (10) Tan (2014) [44]

Twelve articles in the systematic review used the MMSE to diagnose Alzheimer’s disease, 10 gave an MMSE score with the standard deviation. The numbers were extracted and compiled into the plot in Fig. 3, which shows a decreasing progression of MMSE scores from MCI at 21 to severe cognitive impairment at 10.

Eight miRNAs have been found to be significantly deregulated when comparing both control and MCI, and control and Alzheimer’s disease candidates. Two miRNAs are significantly different between MCI and Alzheimer’s disease (193b and 200b) [49, 55]. However, the presence of MCI does not guarantee an Alzheimer disease diagnosis; therefore, the miRNA specific to MCI that develops into Alzheimer’s disease must be extracted.

Four miRNAs were found to be significantly deregulated in two different articles (Table 3). However, only two were consistent between articles (125b and181c) and two were inconsistent (9 and 135a-5p). Both mir-9 and mir-181c have MMSE scores assigned to the two articles, the first article has an MMSE score of 10.5 and the second 15.

**Table 3:** MiRNA found to be consistent and contradictory in serum between different articles

miRNA	First article	Second article
9	↑	↓
125b	↓	↓
181c	↓	↓
135a-5p	↑	↓

Sensitivity and specificity values were extracted from nine articles and are shown in Table 4.

**Table 4:** Sensitivity and specificity values for blood deregulated miRNA

Study ID	Author	No. of patients	No. of controls	Blood component	Sensitivity	Specificity	miRNA profile
1	Wang (2015)	97	81	Plasma	0.90	0.78	mir-107
3	Tan (2014)	105	150	Serum	0.87	0.53	mir-9
					0.81	0.68	mir-125b
					0.75	0.64	mir-181c
4	Tan (2014)	208	205	Serum	0.85	0.71	mir-342-3p
					0.81	0.68	mir-342-3p, -98-5p, 885-5p, -191-5p, 483-3p, -7d-5p.
6	Cheng (2014)	39	59	Serum	0.87	0.77	mir-30e-5p, -101-3p, -15a-5p, -20a-5p, -93-5p, -106b-5p, -18b-5p, -106a-5p, -1306-5p, - 3065, -582-5p, -143-3p, -335-5p, -424-5p, -342-3p, -15b-3p
7	Kumar (2013)	31	37	Plasma	0.20	0.88	mir-545-3p
					0.95	0.53	let-7g-5p
					0.85	0.88	mir-15b-5p
					0.95	0.94	mir-545-3p, -7g-5p, -15b-5p
					0.65	1	mir-142-3p
					0.95	0.76	mir-191-5p
					0.75	0.88	let-7d-5p
8	Leidinger (2013)	142	43	WB	0.92	0.95	mir-7f-5p, -1285-5p, -107, -103a-3p, -26b-5p, - 532-5p, -151a-3p, -161, -7d-3p, -112, -5010.
9	Bhatnagger (2014)	110	123	BMC	0.92	0.96	mir-34a
					0.84	0.74	mir-34c

**Fig. 4:**
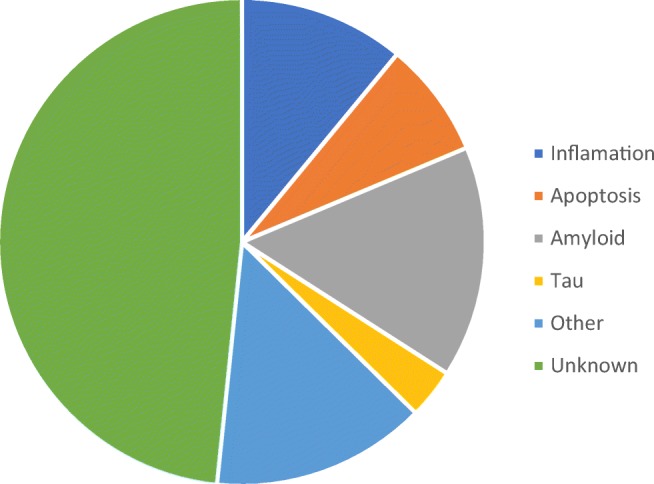
miRNA deregulated in peripheral blood experimentally found targets grouped into inflammation, apoptosis, amyloid, and tau signalling pathways

A further literature study was conducted to establish the role of each blood deregulated miRNA. This is to determine whether the miRNA in the blood are predominately associated with inflammation, amyloid-beta, or tau signalling pathways. Results in Fig. 4 show that 44 miRNAs have unknown targets, 14 from amyloid, 10 from inflammation, 7 from apoptosis, 3 from tau, and 13 from other signalling pathways.

## MiRNA Deregulated in the CSF

Twelve articles were found to contain data for deregulation of miRNA in the CSF; this resulted in 153 deregulated miRNAs. Nineteen miRNAs were found to be deregulated between more than one article; all but 6 had consistent results.

A recent multi-centre study looking at the deregulation of 4 miRNAs in the CSF of Alzheimer patients and found significant differences between results from the three centres [63]. All centres used PCR for analysis and the same RNA isolation procedure. After analysis, the multi-centre study found a significant difference between centrifuged and non-centrifuged samples before freezing and correlations between the PCR cycle threshold (Ct) values and storage time. This highlights the need for detailed standardised procedures.

## miRNA Deregulation in the Brain

Twenty-seven articles were found looking at deregulated miRNA in the brain, corresponding to 250 miRNAs. The search included 13 articles from the temporal cortex [64–76], 6 from the hippocampus [65, 77–81], 8 from the frontal cortex [77, 78, 82–87], 1 from the entorhinal region [81], and 1 the parietal lobe [88].

**Fig. 5:**
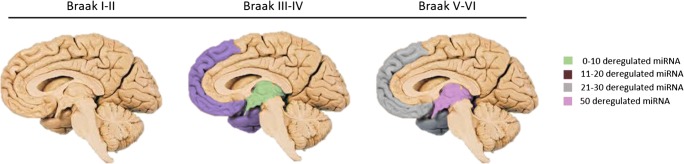
Schematic showing the number of miRNA deregulated in the different areas of the brain

Articles that define the Braak Stage were extracted and split into three groups, Braak Stage I-II, Braak Stage III-IV, and Braak Stage V-VI. Braak Stages I and II are generally used as control cases, 27 miRNAs were deregulated at Braak III–IV and 99 at Braak V–VI, as shown in Fig. 5. Five hundred millilitres of CSF can be absorbed into the blood daily, and damage to the blood brain barrier during Alzheimer’s disease enables exchange of miRNA between the brain and peripheral blood [89]. Therefore, miRNAs deregulated in the blood were cross-referenced against those deregulated at Braak Stage III.

## miRNA Deregulated in the Brain and Blood

All deregulated miRNAs in the peripheral blood were cross-referenced against the miRNA deregulated in the brain. Forty-seven miRNAs are deregulated in both the brain and peripheral blood, 30 of these could be assigned a Braak Stage. From the 30 miRNAs, 10 were found to be deregulated at Braak Stage III; these miRNA are shown in Table 5.

**Table 5:** MiRNA deregulated at Braak Stage III in the brain and in the peripheral blood of Alzheimer patients

miRNA	Brain			Blood		
107	↓	TC	[66]	↓	WB	[41, 61]
				↓	P	
26b	↕	TC	[69]	↓	WB	[41]
30e	↑	H	[78]	↑	EXO	[42, 47]
				↑	S	
34a	↑	H	[76, 78]	↑	BMC	[51, 52]
	↓	TC				
				↓	P	
	↑	FC				
485	↓	FC	[77]	↓	S	[47]
200c	↑	H	[78]	↑	P	[90]
210	↓	H	[78]	↓	S	[59]
146a	↑	H	[78, 80]	↓	P	[51, 57]
	↑	FC		↓	S	
34c	↑	H	[80]	↑	S	[43, 46]
				↑	BMC	
125b	↑	H	[78]	↓	S	[60]

*TC*, temporal cortex; *H*, hippocampus; *FC*, frontal cortex; *WB*, whole blood; *P*, plasma; *S*, serum; *EXO*, exosomes; *BMC*, blood mononuclear cells

Among these, 10 miRNAs that were deregulated both in the brain Braak Stage III and in peripheral blood; 4 miRNAs, namely mir-26b, mir-34a, mir-146a, and mir-125b, were found to be differently deregulated in the two tissues, i.e. upregulated in the brain but downregulated in blood. However, mir-34a was reported to be upregulated in blood mononuclear cells in a study (current reference [52] i.e. Schipper et al).

## Network Analysis of Deregulated miRNA at Braak Stage III in the Brain and Peripheral Blood

The 10 miRNAs found to be deregulated at Braak Stage III and in the blood (Table 5) were imputed into the mirnet online software [91]. Seven of the 10 miRNAs, namely mir-107, mir-26b, mir-30e, mir-34a, mir-210, mir-146a, and mir-125b, resulted in Alzheimer’s disease as at least one of their target diseases from the software analysis. Interestingly, out of these 7 miRNAs that targeted Alzheimer’s disease, 3 miRNAs, namely mir-107, mir-30e, and mir-210, were found to be similarly deregulated in the brain and peripheral blood according to Table 5.

**Fig. 6:**
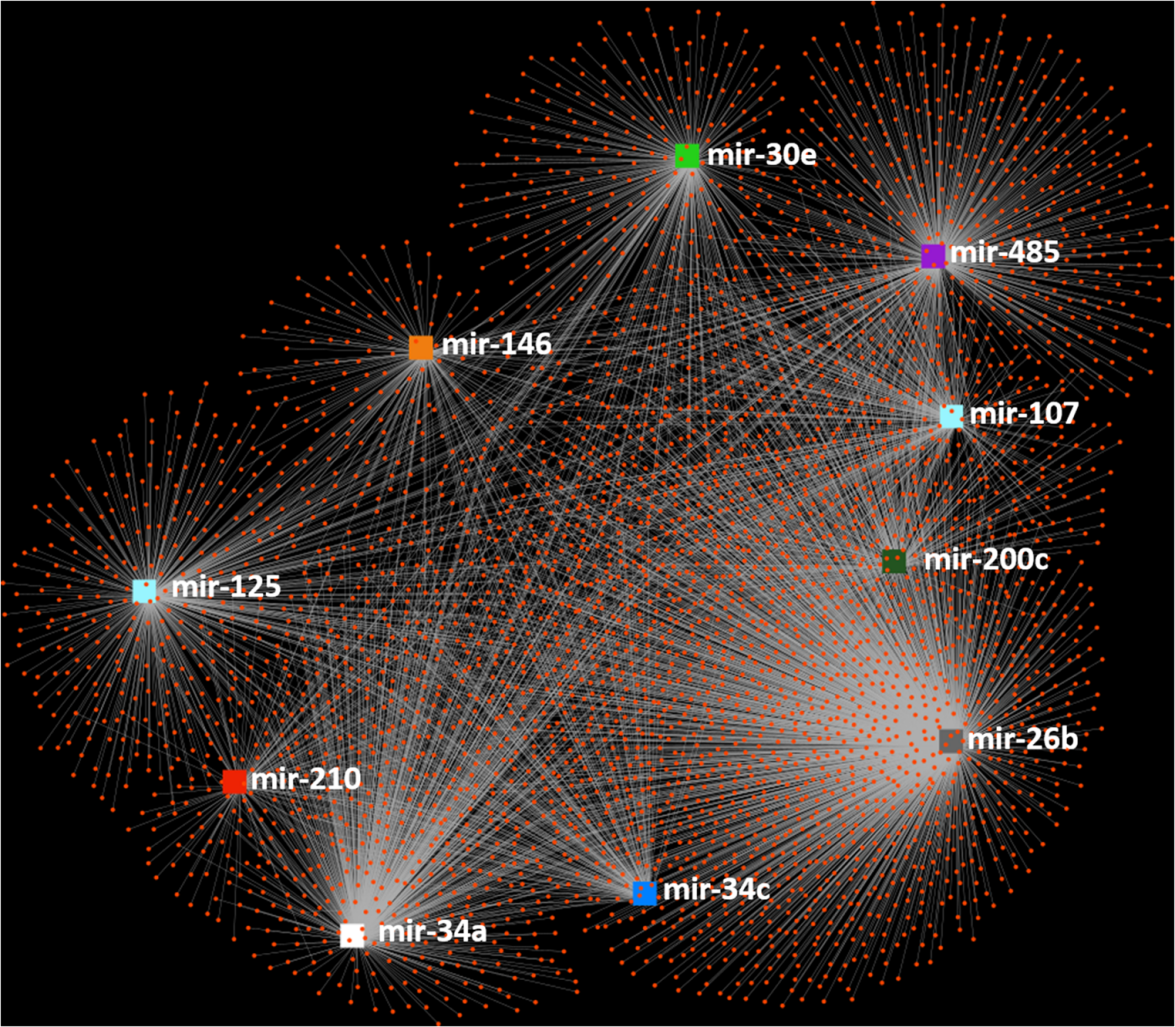
Network diagram extracted from mirnet [91] miRNA’s () targets ()

**Fig. 7:**
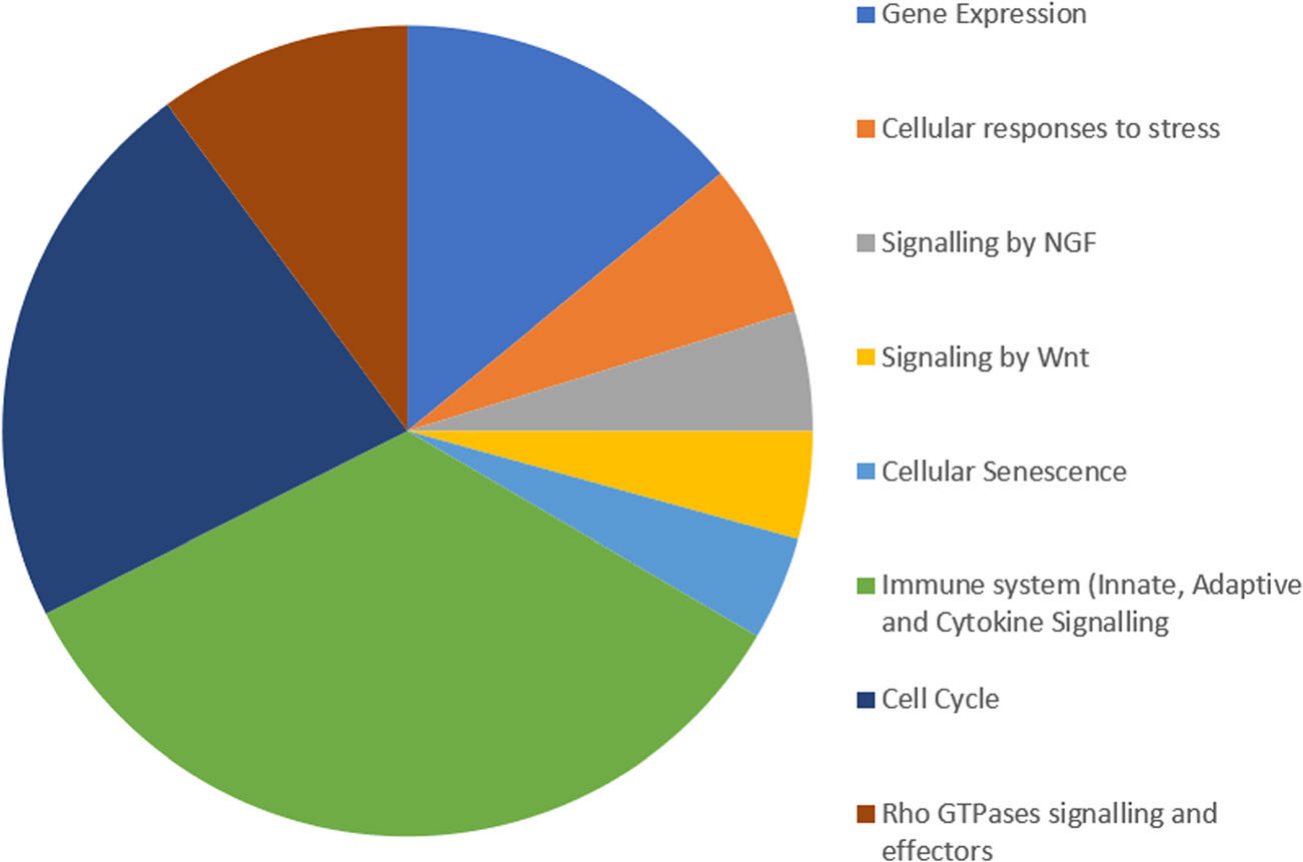
Roles of targets found in network analysis (Fig. 6)

The mirnet online software was also employed to analyse the target genes for the 10 miRNAs listed in Table 5. The resulting network diagram, shown in Fig. 6**,** resulted in 5173 targets associated with the 10 miRNAs. A reactome analysis was conducted using the mirnet software to determine the roles of the target genes. Only statistically significant (*p* ≤ 0.05) groups were then extracted from mirnet. The statistically significant groups with more than 85 target genes are shown in Fig. 7.

Eight different groups are outlined in Fig. 7: immune system (716 targets), cell cycle (469 targets), Rho GTPases (212 targets), gene expression (295 genes), cellular response to stress (130 targets), nerve growth factors (NGF) signalling (100 targets), Wnt signalling (90 targets), and cellular senescence (87 genes).

Chronic inflammation is well reported in the brain during Alzheimer’s disease leading to oxidative stress. Because of this, anti-inflammatory and anti-oxidant agents are being investigated as a disease-modifying therapy [92–94]. Both mir-125b and mir-146a have been connected to neuroinflammation, and they are significantly upregulated by NF-kB, a pro-inflammatory transcription factor [95].

Abnormal expression of cell-cycle proteins have been found in neurons; generally, neurons are post-mitotic [96]. mir-26b has been implicated in cell-cycle regulation through Rb1/E2F and p27/kip1 [69], mir-107 regulates CDK6 [97], and mir-125b can downregulate the cell-cycle inhibitor CDKN2A [98]. mir-34a has also been found to be important in the regulation of the neuronal cell cycle and apoptosis [99].

Rho and its effectors have been linked to amyloid-beta production, as inhibition of Rho-associated kinase was found to reduce cortical amyloid-beta 42 by 33% in mice [100]. Amyloid beta has been found to target Rho GTPases, which may result in changes in the actin cytoskeleton [101]. mir-34a can repress expression of RhoA [102], which is reduced in the post-mortem Alzheimer disease brain [103].

Altered gene expression in the brain between aged control and Alzheimer patients has been documented [104–106].

The increased oxidative stress in the brain during Alzheimer’s disease induces a stress response in the cells, for example the release of IL-6, which is altered in the brain of Alzheimer patients [107]. Cell culture models using neurones have found an upregulation in mir-210 and mir-146a in response to increased ROS [108, 109].

There is a moderate increase in NGF in all brain regions except for the nucleus basalis in Alzheimer’s disease [110]. NGF is a protein, which promotes the growth and survival of cholinergic neurons, which degenerate in the nucleus basalis during Alzheimer’s disease [111]. However, this is not an early pathological event in Alzheimer’s disease as cholinergic neurons in early Alzheimer’s disease (mild cognitive impairment) show no significant difference to patients with no cognitive impairment [112]. Decreased expression of mir-210 has also been found in response to NGF treatment [113].

Various Wnt signalling components are altered in Alzheimer’s disease, for example Dkk1 is increased in the Alzheimer disease brain and is implicated in tau phosphorylation. Some studies have also shown Wnt signalling to be neuroprotective [114, 115]. Mir-107 has been shown to regulate Dkk1; however, this was in osteosarcoma [116].

The presence of cellular stress can induce senescence. Cell culture models have shown that amyloid beta can accelerate cellular senescence [117] and there is an increased number of senescent astrocytes in the brain [118]. mir-125b is a negative regulator of p53 in humans [119]. p53 is implicated in cell-cycle control, apoptosis, DNA, and cellular stress and contributes to cellular senescence [120].

## Conclusion

The review outlines an alternative approach to finding early miRNA biomarkers for Alzheimer’s disease. It utilises miRNA deregulated in the blood during late Alzheimer’s disease and compares to miRNA found to be altered in the brain during early Alzheimer’s disease. However, the literature is riddled with inconsistency. This could stem from technical variations or from limitations in comparability due to differences in a patient’s stage of Alzheimer’s disease (Fig. 3). To improve comparability, Alzheimer patients could be grouped into Braak Stages, and direct comparisons could be made between their pathology and miRNA profile in peripheral blood. Multi-centre comparisons would also benefit from having a standardised analytical protocol, storage time, and quantification method. The review also highlights the possibility of using miRNA deregulated in post-mortem brain samples to identify potential biomarker targets, which is possible due to the higher stability of the miRNAs compared to that of mRNA.
